# The Promise of Single-Domain Antibodies as Ocular Therapeutics: A Narrative Review

**DOI:** 10.3390/ijms27115080

**Published:** 2026-06-04

**Authors:** Thomas Stax Jakobsen, Karoline Kaptain, Kathrine Pedersen, Rikke Lentz Adsersen, Lars Aagaard, Anne Louise Askou, Thomas J. Corydon

**Affiliations:** 1 Department of Biomedicine, Aarhus University, 8000 Aarhus, Denmark; 2 Department of Ophthalmology, Rigshospitalet, 2600 Glostrup, Denmark; 3 Department of Ophthalmology, Regional Hospital Gødstrup, 7400 Herning, Denmark; 4 Department of Ophthalmology, Aarhus University Hospital, 8200 Aarhus, Denmark

**Keywords:** single-domain antibody, nanobody, gene therapy, retinopathy, age-related macular degeneration, eye disease

## Abstract

Single-domain antibodies (sdAbs) are the smallest antigen-binding antibody (Ab) fragments (12–15 kDa) and have emerged as a versatile therapeutic platform. Their compact size, high solubility, stability, and ability to access cryptic epitopes distinguish them from conventional monoclonal Abs (mAbs) and larger Ab fragments. These properties are particularly attractive in ophthalmology, where molecular size, tissue penetration, and formulation constraints critically influence therapeutic performance. This narrative review summarizes the structural features, engineering strategies, immunogenicity considerations, and production platforms of sdAbs, with a focus on ocular applications. Preclinical studies demonstrate promising efficacy in retinal vascular diseases through targeting of VEGFA, ANG2, TNFα, and complement components, as well as in inflammatory and anterior segment disorders. SdAbs can be formatted as multimeric or Fc-fused constructs to extend intraocular half-life or delivered via gene therapy vectors as a sustained intraocular “biofactory” approach. Notably, recent work demonstrates the feasibility of vector-encoded sdAbs targeting complement C3 in vivo. While challenges remain regarding immunogenicity, pharmacokinetics, and regulatory pathways, the approval of several sdAb-based drugs in other fields underscores their clinical potential. SdAbs represent a promising next-generation modality for ocular therapeutics, enabling innovative strategies beyond conventional antibody formats.

## 1. Introduction

Monoclonal antibodies (mAbs) and derived fragments are highly successful drug classes [1]. This is also the case in ophthalmology as illustrated by the available vascular endothelial growth factor (VEGF)-neutralizing agents used for neovascular and exudative eye disease. Bevacizumab (Avastin), used off-label, is a full-length immunoglobulin G1 (IgG1) mAb. Ranibizumab (Lucentis) is an antigen-binding fragment (Fab). Brolucizumab (Beovu) is a single-chain fragment variable (scFv). Faricimab (Vabysmo) is a full-length bispecific mAb binding vascular endothelial growth factor A (VEGFA) and ANG2. Aflibercept (Eylea) is a fusion protein in which portions of human VEGF receptors 1 and 2 are fused to the constant fragment crystallizable (Fc) portion of IgG1 [2]. It neutralizes VEGFA but also VEGFB and placental growth factor.

**Figure 1:**
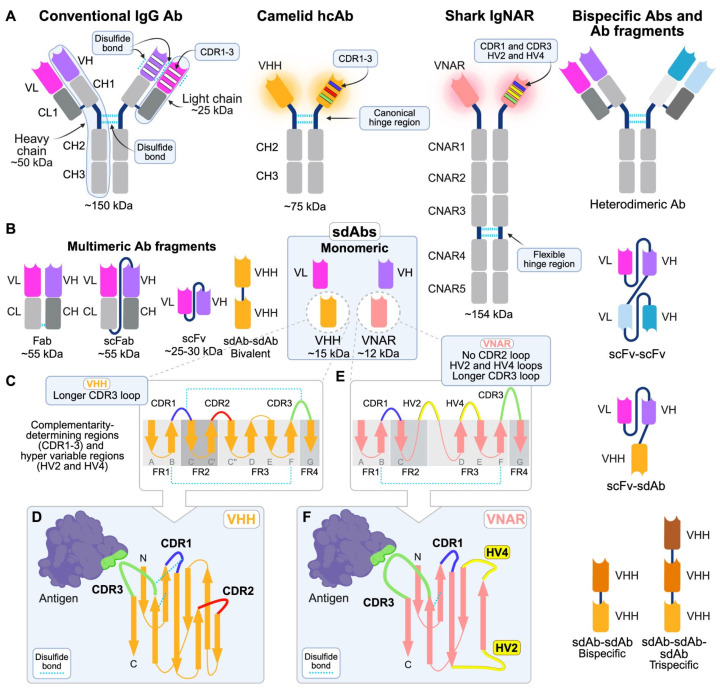
Antibodies, derived fragments, and distinct structural architecture of single-domain antibodies. (**A**) Structural representation of conventional IgG Ab, camelid hcAb, shark IgNAR, and bispecific Abs and Ab fragments, including scFv-scFv, scFv-sdAb, sdAb-sdAb, and sdAb-sdAb-sdAb. CDRs and HVs are depicted in the variable domains. Disulfide bonds are depicted in light blue (dashed). Molecular size of selected structures is shown below. (**B**) Structural illustration of monomeric and multimeric Ab fragments including sdAbs (VHH, VNAR, VH, and VL), bivalent sdAb, scFab, Fab, and scFv. (**C**,**D**) Two-dimensional representation of the VHH structure illustrating the canonical nine immunoglobin β-strands comprising the framework region (FR1–FR4), CDR1 (dark blue), CDR2 (red), and CDR3 (green). Disulfide bonds are depicted in light blue (dashed). (**E**,**F**) Two-dimensional schematic of the VNAR structure illustrating the seven immunoglobulin β-strands that form the framework regions (FR1–FR4), together with the complementarity-determining regions CDR1 (dark blue) and CDR3 (green), and the hypervariable loops HV2 (yellow) and HV4 (yellow). In addition to the HV2 and HV4 regions, the VNAR framework is characterized by the absence of C′ and C″ β-strands and the conventional CDR2 region. Disulfide bonds are indicated in light blue (dashed). Ab, antibody; CDR, complementarity-determining region; C, carboxy-terminus; CH, constant domain of heavy chain of IgG; CL, constant domain of light chain; CNAR, constant domain of IgNAR; Fab, antigen-binding fragment; FR, framework; hc, heavy chain; HV, hyper variable regions; IgNAR, shark immunoglobulin new antigen receptor; N, amino-terminus; scFab, single-chain antigen-binding fragment; scFV, single-chain fragment variable; sdAb, single-domain antibody; VH, variable heavy domain; VHH, variable heavy domain of heavy chain; VL, variable light domain. Inspired by Hoey et al. [25], Juma et al. [26], and Evers et al. [27]. Created in BioRender. Aagaard, L. (2026) https://BioRender.com/nzkm1ev (accessed on 1 June 2026).

Full-length mAbs are large (~150 kDa) proteins consisting of two heavy and two light chains containing constant and variable domains: the heavy chain has one variable domain (VH) connected to three constant domains (CH1-3); the light chain has one variable (VL) and one constant domain (CL) (Figure 1A). The first therapeutic mAbs were murine, but immunogenicity has since been reduced by the creation of chimeric, humanized, or fully human mAbs [1]. The clinical application of full-length mAb continues to expand [3] and efficient bispecific Ab platforms enable dual targeting [4,5] (Figure 1A). However, their size may limit tissue penetration and access to certain epitopes. The Fc-region is potentially immunogenic, although engineering strategies have been developed to silence Fc-mediated immune activation [6]. Furthermore, some of these limitations can be overcome by using antibody fragments such as Fabs, single-chain Fabs (scFabs), and scFvs (Figure 1B), which also facilitate recombinant production and the use of low-cost non-mammalian production systems [7,8]. Full-length mAbs, Ab fragments, and ligand-binding receptor domains can be combined to create an extensive toolbox of engineered molecules [7].

A special group of Ab fragments are single-domain Abs (sdAbs) or “nanobodies”, which are the smallest Ab fragments (12–15 kDa). Their unique structural properties confer several advantages allowing them to compare favorably with other Ab fragments such as scFv [9]. Accordingly, they have been used for a range of scientific, diagnostic, and therapeutic purposes [10,11,12], and recent years have seen the advent of clinically approved sdAb therapies: the bivalent sdAb caplacizumab binding von Willebrand factor is FDA approved for acquired thrombotic thrombocytopenic purpura [13]. The trimeric sdAb ozoralizumab binds TNFα and human serum albumin and has been approved for rheumatoid arthritis in Japan [14]. The homodimeric fusion protein envafolimab consisting of a sdAb binding programmed death-ligand 1 and the human IgG1 Fc-region has been approved for solid tumors in China [15]. SdAbs can also substitute scFvs in chimeric antigen receptor T-cell therapy: ciltacabtagene autoleucel was FDA approved for relapsed or refractory multiple myeloma. It uses two sdAbs to bind different epitopes of the B-cell maturation antigen for improved avidity [16]. Hence, sdAbs have emerged as a promising therapeutic platform with potential applications across a broad spectrum of diseases.

This review surveys the developments in therapeutic application of sdAbs in ophthalmology. A narrative review format was chosen to accommodate the scope of the review. However, all relevant preclinical studies were identified through a systematic search in PubMed (Supplementary Materials). Preclinical animal studies are summarized in Table 1.

## 2. Structure, Design, and Properties of sdAbs

The basis for naturally derived sdAbs was the discovery of camelid heavy-chain-only antibodies (hcAb) [17] and later the shark immunoglobulin new antigen receptor (IgNAR) [18], which both depend on a single domain for antigen recognition.

The antigen-binding domain of camelid hcAbs is known as the variable heavy domain of heavy chain (VHH). Antigen binding is primarily mediated by the three complementary-determining regions (CDRs) of VHH with a typically elongated CDR3 loop compared with those of VHs in conventional IgG Abs. Combined with the small size, this enables binding to concave epitopes, including active sites of enzymes or receptor domains [19], thereby increasing the range of druggable targets. The presence of hydrophilic amino acids in the framework region 2 (FR2) that normally is buried in the VH-VL interface leads to high water solubility and reduces aggregation, and their small, single-domain nature combined with, e.g., non-canonical disulfide bonds, provides a compact and stable structure [12] (Figure 1C,D).

The antigen-binding domains of IgNAR, denoted variable new antigen receptors (VNARs), consist of a classical immunoglobulin fold but lack part of the FR2-CDR2 region making them the smallest antigen-binding Ab-derived moiety. It shares several characteristics of VHH with high stability and solubility and an elongated CDR3 loop. Antigen-binding is facilitated by the remaining CDR1 and CDR3 loops and two additional hypervariable loops (HV2 and HV4) [19,20,21] (Figure 1E,F). The four antigen-binding loops, together with variation at the sequence level, contribute to the extensive diversity in binding. The greater similarity of VHH to human VHs, robust hcAb responses following immunization, and the practical advantages of working with llamas compared with sharks have made VHHs the dominant sdAb platform although VNARs emerge as a competitive platform [21].

SdAbs may also be engineered from human variable domains (Figure 1B). In particular, sdAbs engineered from the VH of human IgG are being explored, and the concept of sdAbs initially derived from the preparation of VH repertoires capable of antigen binding [22]. VL sdAbs can also be created [23]. Human-derived VLs or VHs theoretically provide reduced immunogenicity but lack some of the advantageous properties inherent to camelid and shark-derived sdAbs. The CDR3 loop is shorter and with reduced flexibility, and they must be modified to achieve comparable solubility, stability, and affinity. These features are often highly dependent on the CDR regions, which may complicate development [24].

The small size of sdAbs and other isolated Ab fragments limits tissue retention and systemic half-life, which is further reduced by the lack of an Fc region required for neonatal Fc receptor-mediated recycling. However, retention can be readily improved by fusion to human Fc or an albumin-binding moiety. Fusion to a non-silenced Fc provides Ab effector functions that are desirable for some applications [7]. SdAbs can also be combined into multivalent or specific constructs or can be used as building blocks for complex formats [7] (Figure 1A).

### 2.1. Development and Production of sdAbs

**Figure 2:**
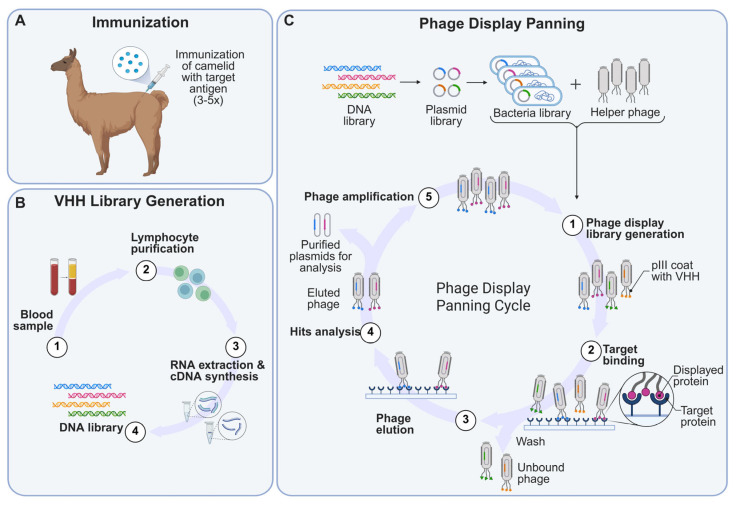
Platform for efficient discovery and development of antigen-specific sdAbs from VHH immune libraries. (**A**) Immunization. Construction of a VHH immune library from immunized camelids (e.g., llamas or alpacas), generating a large and diverse repertoire (10^7^–10^9^ clones) of VHH-encoding sequences. (**B**) Following immunization, peripheral blood lymphocytes are isolated and VHH sequences are recovered from B cells and cloned into phagemid vectors. Owing to clonal expansion and affinity maturation, these libraries are enriched in high-affinity, antigen-specific binders. (**C**) Phage display and panning. Phagemids are propagated in *Escherichia coli* and packaged into filamentous phage particles upon helper phage infection, enabling display of VHH domains fused to the pIII coat protein. This genotype–phenotype linkage allows efficient selection of antigen-specific VHHs by iterative panning against immobilized target antigens, with increasing stringency to enrich high-affinity binders. Selected clones are screened (e.g., by phage ELISA), sequenced, and recombinantly expressed in suitable systems, including cost-effective bacterial or yeast platforms, enabling downstream development for therapeutic, diagnostic, and bioengineering applications. pIII, protein III (from filamentous bacteriophages); VHH, variable heavy domain of heavy chain. Created in BioRender. Larsen, M. (2026) https://BioRender.com/1aw17ea (accessed on 1 June 2026).

Well-established protocols exist for the production of high-specificity VHHs following camelid immunization and subsequent generation of libraries for display selection (Figure 2) [24,28]. Similarly, VNARs can be generated following shark immunization. Such immune libraries efficiently provide high affinity sdAbs due to the clonal expansion and affinity maturation occurring during the natural immune response [21]. Potential challenges related to working with camelids can be overcome by using transgenic mice as recently described with the LamaMice [29].

As an alternative to immune libraries, naïve or synthetic libraries can be constructed. These approaches avoid the need for animal immunization. However, naïve libraries lack the antigen-driven immune response and affinity maturation processes, which may limit library diversity and result in lower frequencies of high-affinity binders. In contrast, synthetic libraries completely omit the natural immune selection in vivo, increasing the risk of generating variants with suboptimal structural properties [24,30]. Nonetheless, synthetic libraries facilitate the construction of sdAbs based on specific scaffolds and rational diversification strategies as demonstrated in numerous publications and discussed at length in previous reviews [24,31,32]. Recent advances in synthetic libraries and development of sdAbs incorporate in silico and artificial intelligence-assisted design strategies. Moreover, machine learning models and large language models are increasingly being explored as data-driven strategies for development of novel sequences and are expected to reduce the reliance on animal immunization in the future [24]. However, these approaches are limited by the availability of training datasets and risk of potential model bias, emphasizing the continuous need for experimental validation of predicted sequences. Thus, immune libraries remain the most widely used method for sdAb discovery [11].

Structural features of sdAbs confer several advantages in library construction and display-based selection compared with mAbs and scFv fragments [9]. The monomeric nature of VHHs and VNARs eliminates the need for heavy–light chain pairing, eliminating mispairing issues, while offering enhanced stability and improved folding properties [21]. In addition, VHHs can be efficiently humanized while maintaining stability and antigen-binding properties. This is due to their high sequence similarity with the human VH3 domains [33,34]. However, certain residues are critical for solubility and binding properties, and these residues must be carefully preserved [33]. SdAbs and derived immunoreagents can be recombinantly expressed using a range of different production systems [35]. This includes inexpensive non-mammalian systems such as bacteria and yeast, which is another considerable advantage.

### 2.2. Immunogenicity

A pertinent question is the potential immunogenicity of the different sdAbs compared with fully humanized mAbs and mAbs fragments. Camelid VHHs have high similarity to human VHs. The up to 95% identity of the framework regions [36] is comparable to that achieved through antibody humanization. However, differences in certain exposed moieties are potentially immunogenic. This includes the normally buried interface between VH and VL, the distinct conformation of the elongated CDR3 loop, and structural changes introduced by non-canonical disulfide bridges. Features favoring low immunogenicity include their small size, rapid systemic clearance, and their resistance to forming immunogenic aggregates [37].

Immunogenicity can theoretically be reduced through humanization, i.e., the replacement of non-human with human sequences in framework regions. This approach is routinely applied for the clinical development of VHHs. However, such modifications may compromise solubility and stability, which may induce immunogenic aggregates. Alternatively, the synthetic human VH or VL sdAb formats can be used, but the immunogenic features of the exposed VH-VL interface are still present, and unusual amino acid composition within the CDR regions can be introduced. Hence, it is not evident that they are less immunogenic [37]. There are also established protocols for humanization of VNARs [21,37].

Limited in vitro studies have investigated immunogenicity to sdAbs: Non-humanized VHH demonstrated marginal capacity to activate dendritic cells or to induce T-cell proliferation in one study [38]. Only a single patient had low levels of pre-existing anti-drug Abs (ADAs), which increased marginally following systemic exposure when this VHH was tested for diagnostic purposes [38]. Other clinical trials, mostly using humanized VHHs, have shown low levels of immunogenicity [37]. Higher levels of pre-existing ADAs have been observed for both a VH antagonizing TNFR1 (GSK1995057) [39,40] and a tetravalent humanized camelid VHH binding DR5 (TAS266) [41], which was associated with cytokine release syndrome and hepatotoxicity, respectively. The exact determinants remain uncertain, and it should be noted that fully human mAbs still can induce ADAs. SdAbs also emerge as a promising alternative to smaller Ab-derived fragments such as scFabs and scFvs. These non-native single-chain formats necessitate the design of an appropriate linker that may serve as an immunogenic epitope, and they are often less stable and prone to aggregation contributing to their immunogenic potential [9]. Notably, high rates of ocular inflammation were observed following intravitreal delivery of the scFv brolucizumab, which was related to such features [42].

Additional strategies to reduce immunogenicity are removal of CD4+ T-cell epitopes (TCEs), which is denoted de-immunization, or introduction of regulatory TCEs, which is denoted tolerization [43], but they currently remain unexplored. There is also a lack of investigation into systemic and local immune responses following intraocular delivery of sdAbs, which will be essential for therapeutic development for posterior segment eye disease.

## 3. Therapeutic Applications of sdAbs in Ocular Disease

**Figure 3:**
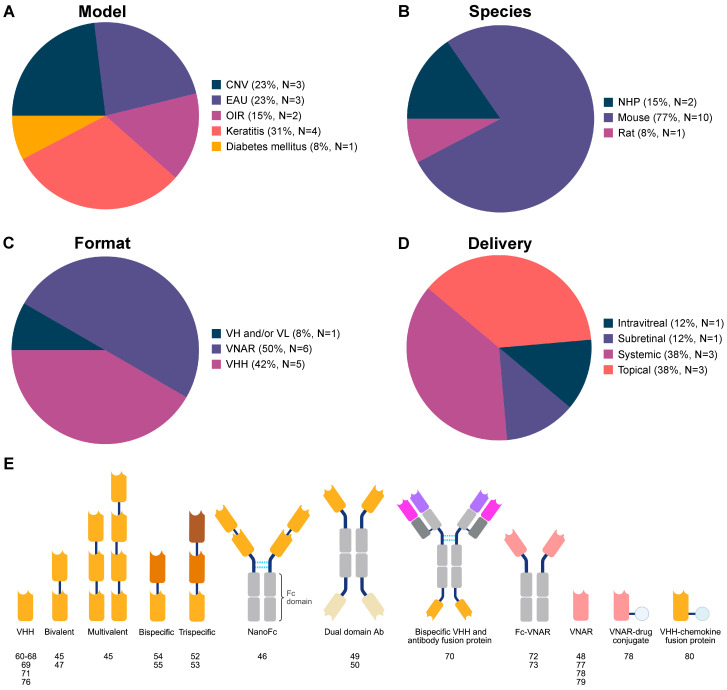
Summary of preclinical ocular studies and structural features of applied single-domain antibodies. (**A**–**D**) Pie charts illustrating key observations (related to model, species, format, and delivery), extracted from the preclinical ocular studies summarized in Table 1. N represents the number of studies. (**E**) Schematic representation of the structural architecture of sdAbs used in the studies listed in Table 1 and described in Section 3 and Section 4. Numbering corresponds to the studies referenced in these sections. CNV, choroidal neovascularization; EAU, experimental autoimmune uveitis; Fc, fragment crystallizable; NHP, non-human primates; OIR, oxygen-induced retinopathy; VH, variable heavy domain; VHH, variable heavy domain of heavy chain; VL, variable light domain; VNAR, variable new antigen receptors. Created in BioRender. Bartholin, C. (2026) https://BioRender.com/qkvkc4e (accessed on 1 June 2026).

Preclinical therapeutic sdAb studies in ophthalmology (Table 1) encompass several disease models. Most investigate the therapeutic utility in posterior segment disease using established models such as the laser-induced choroidal neovascularization (CNV) model [44], but a significant portion explores the utility in microbial keratitis (Figure 3A). The studies are dominated by rodent models (Figure 3B) and are almost equally divided between VNAR and VHH formats, while only a single study uses a combined VH and VL construct (Figure 3C). For posterior segment disease systemic and topical delivery dominates (Figure 3D) illustrating an interest in harnessing the pharmacokinetic properties of sdAbs for non-invasive drug delivery.

### 3.1. Retinal Vascular Disease

A major focus of sdAb studies has been retinal vascular diseases (Figure 3A). The dominating target for exudative retinal diseases such as diabetic macular edema and neovascular age-related macular degeneration (nAMD) is VEGFA. Several VEGF-binding sdAbs have been developed. For ocular application, the use of multimeric sdAbs or fusion to an Fc domain to increase ocular retention are dominating strategies (Figure 3E). Multivalent sdAbs binding VEGF with optimized linkers demonstrated increased VEGF binding affinity and in vitro inhibition of the VEGF/VEGF receptor (VEGFR) pathway compared with the monovalent forms [45]. Fusion of such multivalent sdAbs to an Fc domain (“nanoFc”) further enhanced VEGF binding and effect in bioassays, while showing high stability and the ability to be formulated in high molar concentrations [46].

Recently, a bivalent humanized VHH (Figure 3E) against VEGFA was designed to be delivered topically using a proprietary penetratin analog formulation. Both topical delivery and intravitreal delivery attenuated pathological features in a murine oxygen-induced retinopathy (OIR) and a non-human primate (NHP) laser-induced CNV model [47]. Previously, a VEGFA_165_-binding VNAR has been shown to enter the aqueous humor in rabbits following topical delivery and to reduce endothelial cell proliferation in a murine OIR model [48].

A more complex design based on VH and VL sdAbs was presented in an earlier study. These “dual domain Abs” are dimeric with each monomer containing two distinct VEGF-binding sdAbs attached via linkers to a human IgG1 Fc domain (Figure 3E). Delivery using microparticles based on a PolyActive™ hydrogel co-polymer resulted in prolonged release sufficient to inhibit leakage in the laser-induced CNV model [49,50].

ANG2 is another promising target and dual ANG2/VEGFA targeting with the bispecific mAb faricimab is approved for retinal vascular disease [51]. Intravitreal delivery of the humanized trimeric VHH construct neutralizing VEGF and ANG2 (BI-836880) has been tested in a phase I clinical trial in nAMD patients [52,53], and preclinical data supports vitreous half-life extension through the inclusion of an albumin-binding VHH [52]. Encouragingly, intravitreal delivery of BI-836880 was well-tolerated in the clinical trial [53].

VEGF- and TNFα-binding VHH sdAbs have also been combined. The bispecific VHH-neutralizing VEGFA and TNFα reduced angiogenic signaling through VEGFR2 and release of inflammatory cytokines in the NF-κB/TNF-α pathway in vitro [54]. Another bispecific VEGF- and TNFα-neutralizing VHH further demonstrated the ability to reduce TNF-α and VEGF-A levels in retinal tissues of diabetic mice and reduced retinal neovascularization in streptozotocin-induced diabetic mice following topical delivery [55].

SdAbs binding VEGFA and other angiogenic molecules are potential candidates not only for nAMD and diabetic macular edema but may also have therapeutic roles in CNV caused by other chorioretinal diseases and retinal neovascularization in, e.g., proliferative diabetic retinopathy and retinopathy of prematurity.

### 3.2. Inflammatory Pathways

Additional important targets include inflammatory pathways. Notably, complement dysregulation is strongly associated with AMD [56] and complement inhibition is an established therapy for geographic atrophy following FDA approval of the pegylated anti-C3 and C3b peptide pegcetacoplan [57] and the pegylated C5-targeting RNA aptamer avacincaptad pegol [58,59]. A range of different complement-inhibiting VHHs have been developed [60,61,62,63,64,65,66,67,68] enabling targeted or general suppression of the complement cascade. The first study to explore a therapeutic effect in vivo used subretinal delivery of a lentiviral (LV) vector encoding a VHH inhibiting the alternative and terminal pathway by binding to C3 [69]. Efficient in vivo expression correlated with suppression of laser-induced CNV formation proving the potential for vector-encoded sdAb delivery to the eye.

Ozoralizumab is an approved VHH-based TNFα-inhibitor, and one may suppose that it as other systemic TNFα inhibitors will be effective for the ophthalmic indication refractory non-infectious uveitis. As described above, bispecific VEGFA- and TNFα-binding sdAbs have been developed. A bispecific TNFα-binding VHH and anti-IL-6 antibody fusion protein (Figure 3E) has been designed for the treatment of inflammatory retinal diseases [70] illustrating the potential use of sdAbs in multi-targeting engineered formats.

Systemic delivery of a VHH neutralizing signal transducer and activator of transcription 3 (STAT3) has been evaluated in an experimental autoimmune uveitis (EAU) model [71]. Notably, targeting of this intracellular protein suppressed EAU by inhibiting expansion of pathogenic Th17 cells. A Fc-VNAR binding induced co-stimulatory ligand (ICOSL) also suppressed EAU following systemic delivery [72]. In a Lewis rat EAU model, systemic delivery of this Fc-VNAR against ICOSL was less effective than Fc-VNAR neutralizing TNFα [73], which significantly ameliorated clinical signs, histologic changes, and aqueous protein levels compared with vehicle.

### 3.3. Molecular Chaperones and Allosteric Modulators

A special application of sdAbs or combined molecules is their use as molecular chaperones or allosteric modulators. This is facilitated by their extraordinary cytosolic stability, which allows them to function as “intrabodies” [74], particularly following optimization [75]. Interesting work demonstrates that specific VHHs can modulate the photoactivation of rhodopsin and prevent disease-associated rhodopsin misfolding [76], which could be a promising strategy for certain inherited retinal diseases.

### 3.4. Anterior Segment Disease

A significant portion of studies have explored the utility of sdAbs for anterior segment disease in the form of microbial keratitis. Topical application of a VNAR directed against dectin 1 reduced inflammation in a mouse model of fungal keratitis [77]. Similarly, a VNAR against β-glucan alone or conjugated with natamycin attenuates corneal injury due to fungal keratitis [78,79]. Furthermore, a VHH–chemokine fusion protein showed therapeutic efficacy in a mouse model of *Acanthamoeba* keratitis [80]. The use of sdAb–drug conjugates or cytokine-fusion proteins (Figure 3E) exemplifies the ability to sdAbs for directed drug delivery.

## 4. Ocular Delivery and Pharmacokinetic Properties

### 4.1. Intravitreal Delivery

Intravitreal delivery is the dominating delivery route for posterior segment diseases as illustrated by the routine use of intravitreal anti-VEGF injection for exudative eye disease. The therapeutic durability of intravitreally injected drugs depends on the potency and achievable concentration as well as the intraocular half-life of the compound. Recent years have clearly proven the therapeutic benefit of high-molar-dose VEGF inhibitors [51,81,82]. However, the delivered dose is limited by small injection volumes and hence the solubility and stability of the compounds. As noted above, these key features of sdAbs are also important immunogenicity determinants. The alternative strategy to increase half-life through structural modifications must be balanced against potential reductions in diffusibility and target engagement.

Ocular pharmacokinetics of monomeric sdAbs have been reported in rabbits with an elimination half-life of about 3 days following intravitreal delivery [83], but not in humans or large animal models as for current therapeutic formats [84,85]: as globular proteins their vitreous half-life correlates well with the molecular weight and hydrodynamic radius [84]. Monomeric sdAbs are expected to have a slightly shorter half-life than current therapeutic formats, which may limit durability. Half-life extension can be achieved using multimeric sdAbs or sdAb-fusion proteins, but this may also negate the advantages associated with improved tissue penetration and limit achievable molar doses. However, a multivalent sdAbs-Fc fusion protein (Figure 3E) could be formulated in high molar concentrations [46]. As noted above, the trispecific sdAb (BI-836880) binding VEGF, ANG2, and albumin is the furthest in development [52,53]. The binding to endogenous vitreous albumin [52] or co-administered albumin [86] extends intraocular half-life. Albumin-binding sdAbs may be used as a general principle to extend half-life [87]. Several alternatives such as PEGylation have been explored for other formats and are promising areas for further research.

The small size of sdAbs may facilitate delivery from extended-release platforms although the only study investigating slow-release from a hydrogel polymer used the complex “dual domain Ab” (Figure 3E) design described above [50]. Similarly, the high solubility and stability of sdAbs make them ideal candidates for port delivery systems that currently rely on the Fab fragment ranibizumab [88]. Polymorphic VHH crystals have also been described as a strategy for slow-release intravitreal delivery [89].

An argument for the use of small Ab fragments is increased tissue penetration although it is still uncertain whether the effect of, e.g., intravitreal anti-VEGF agents, mainly relies on sequestration in the vitreous or depends on diffusion through the retina. The internal limiting membrane is a major barrier. Quantitative comparisons are lacking but current anti-VEGF formats permeate the retina [90,91,92]. The retinal pigment epithelium (RPE) and Bruch’s membrane may further limit action in the choroid as they both demonstrate selective permeability to Abs and Ab fragments in vitro [93,94]. Hence, sdAbs may be uniquely suited to target disease processes in the choroid, but the question remains to be addressed experimentally. Similarly, the potential for improved penetration following suprachoroidal delivery is an intriguing possibility.

### 4.2. Topical Delivery

Topical delivery using eye drops is the obvious solution for corneal disease, but topically delivered antibody fragments can also penetrate the cornea to reach the anterior chamber [95] and vitreous [96]. However, they do not typically reach therapeutic concentrations in the retina following topical delivery in large animals. Hence, ongoing efforts to develop efficient carriers of drugs to the posterior segment following topical delivery [97] and recent preclinical studies support the clinical relevance of topically delivered sdAbs.

A dimeric sdAb targeting TNF-α and VEGF-A decreased retinal levels of both factors in diabetic mice and reduced pathological neovascularization and retinal structural damage [55]. Ocular penetration is clearly different in rodents and large animals, and the continuous suppression of CNV in NHPs using topical delivery of an anti-VEGFA VHH in a proprietary penetratin analog formulation [47] is thus particularly convincing. Topical penetration into the aqueous humor of rabbits was demonstrated for an anti-VEGFA VNAR that could inhibit endothelial cell proliferation in a murine OIR model [48]. Improved topical penetration in mice with debrided corneal epithelium has been described for VNAR and Fc-VNAR compared with mAb [72]. On the other hand, ocular pharmacokinetic studies in rabbits using topical, subconjunctival, and intravitreal delivery of a TNFR1-binding VH (GSK1995057) showed limited penetration into the aqueous humor following topical delivery and no penetration into the posterior segment [83]. Hence, the clinical utility of topically delivered sdAbs for posterior segments disease remains to be proven in clinical studies.

### 4.3. Gene Therapy-Based Delivery of Single-Domain Antibodies

Gene therapy has placed ophthalmology at the forefront of therapeutic development [98]. Efforts are ongoing to expand on the range of eye diseases treatable by gene therapy. The dominating adeno-associated viral vectors (AAVs) can be used as a versatile platform for delivery of protein therapeutics supporting gene agnostic strategies for inherited retinal degenerations or “biofactory” approaches for common retinal diseases such as AMD. Expressed proteins include neurotrophic factors such as pigment epithelium-derived factor [99,100], but as for intravitreally injected therapeutics, Ab fragments and Fc-fusion proteins dominate the developmental pipeline with AAV delivery of transgenes encoding aflibercept [101] and an anti-VEGF Fab [102] in phase III clinical trials [103].

**Figure 4:**
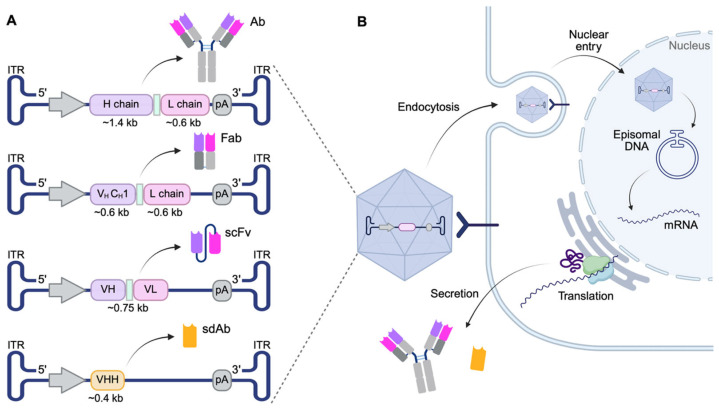
Gene therapy-based delivery of vector-encoded antibodies, derived fragments, and sdAb. (**A**) AAV vectors encoding full length Ab, Fab-fragment, scFv and sdAb, respectively. The distinct chains are encoded from a single expression cassette and divided by a linker or a 2A peptide (light green boxes). Gray arrows indicate promoters. (**B**) The transduction pathway of AAVs. AAVs are internalized through endocytosis, followed by entry into the nucleus, where the DNA remains as episomal DNA for continuous transcription. Following translation and folding, the functional antibodies or antibody derivatives are secreted. AAV, adeno-associated virus; Fab, antigen-binding fragment; H, heavy; ITR, inverted terminal repeat; L, light; mRNA, messenger RNA; pA, poly Adenylation site; scFv, single-chain fragment variable; sdAb, single-domain antibody; VH, variable heavy domain; VHH, variable heavy domain of heavy chain; VL, variable light domain. Created in BioRender. Aagaard, L. (2026) https://BioRender.com/0cfp8lw (accessed on 1 June 2026).

SdAbs seem uniquely suited for vector delivery also in relation to other Ab fragments (Figure 4). Efficient production of full-length Abs following delivery of the encoding transgene requires appropriate folding and assembly involving disulfide linkage as well as N-terminal glycosylation [104,105]. Expression of sdAbs and other fragments are expected to be more efficient, and a 100-fold higher expression of sdAbs compared with mAbs has been observed following AAV delivery of the transgene [106,107]. This is crucial for efforts to reduce vector doses and hence minimize related toxicities. Vector delivery of fragments (or Fc-fusion proteins) accordingly dominates preclinical and clinical development across medical fields [108,109].

AAV delivery of sdAbs or sdAb-fusion proteins is an established strategy for non-ocular diseases [110,111,112]. In the eye, sdAb expression has been shown intracellularly in neonatal mice following plasmid delivery [75]. Recently, an LV vector has been used to deliver a transgene encoding a VHH targeting complement component C3, representing the first demonstration of vector-mediated sdAb expression for therapeutic use in the eye [69]. Following subretinal injection robust expression in RPE cells and secretion into the retina were observed. This correlated with therapeutic efficacy in the laser-induced CNV model. This underscores the potential of vector-delivered sdAbs as a biofactory approach for retinal diseases both on their own or as part of combination gene therapy [103]. SdAbs can additionally be used to alter the tropism of AAVs [113] or serve as targeting moieties for different nanocarriers [114]. Importantly, the continuous expression negates the challenges related to short intraocular retention, while retaining the advantage of excellent tissue penetration and rapid systemic clearance of sdAbs. However, long-term expression strategies also have important caveats due to their irreversible character. The identification of safe dose ranges and the immunogenicity of the expressed compounds become even more important. Furthermore, the long-term safety of sustained complement or cytokine pathway modulation must be considered.

## 5. Towards Clinical Application and Further Directions

Single-domain antibodies (sdAbs) possess several qualities that render them highly attractive for therapeutic applications in ophthalmology, particularly when delivered via gene therapy vectors in a biofactory-based approach enabling sustained intraocular production. Their ability to bind concave epitopes expands the range of druggable targets, and their solubility and stability offer important advantages in terms of safety, intracellular targeting, and delivery to the eye. SdAbs may be used on their own or be used for sophisticated applications through engineered constructs (Figure 1A and Figure 3E).

However, there is a well-established pipeline for the design and production of mAbs, supported by mature regulatory pathways, and mAbs exhibit excellent developability and stability compared with many engineered formats [115]. Humanization of sdAbs remains largely empirical, and uncertainties persist regarding both their immunogenic potential and the structural determinants underlying it. Further refinement of the developmental pipeline is expected to be supported by advances in computational tools [24], which in the future also may include quantum computing-based simulations.

Expiration of key patents [116] and the approval of the first sdAb drugs in recent years offer a promise of new sdAb-based therapies for clinical use also within ophthalmology. However, while the preclinical findings are encouraging, further studies addressing long-term efficacy, immunogenicity, toxicology, and clinical translation are required before broad therapeutic application can be established.

**Table 1:** Summary of preclinical studies investigating sdAb-based therapies in ophthalmology.

Study (PMID)	Animal Species	Model	Nb Structure	Target	Delivery Route	Dose	Target Efficacy	Safety
Adamson et al. 2016 [50]	Cynomolgus monkey	LI-CNV	VH and VL fused to Fc domain (dual domain Ab)	VEGF-A	Intravitreal	1304 μg loaded in 6.0 mg microparticles 16 μg and 160 μg naked	Prevention of grade IV CNV lesions. Sustained intraocular exposure with therapeutic efficacy for up to 6 months using microparticle delivery.	Microparticle-related ocular inflammation, severe cases associated with ADAs Microparticle migration to anterior chamber and delayed polymer degradation
Kovaleva et al. 2017 [72]	Mice (C57BL/6)	EAU	Fc-VNAR	ICOSL (A5-Fc)	Systemic (i.p.)	10 mg/kg daily, day 1 to 14	Reduced ocular inflammation and histopathology scores comparable to cyclosporine A.	No dedicated immunogenicity or toxicology assessment
Camacho-Villegas et al. 2018 [48]	Mice (C56BL/6)	OIR	VNAR	VEGF-A	Topical	0.1 μg/mL eye drops every 6 h for 7 days	Reduced retinal endothelial proliferation (~30%).	No dedicated immunogenicity or toxicology assessment
Pepple et al. 2019 [73]	Lewis rat	EAU	Fc-VNAR	TNF α (S17-Fc) ICOSL (A5-Fc)	Systemic (i.p.)	20 mg/kg, day 8, 10 and 12	S17-Fc reduced OCT scores, histologic inflammation, and aqueous protein levels comparable to dexamethasone. A5-Fc showed a non-significant trend toward disease reduction.	No dedicated immunogenicity or toxicology assessment
Mbanefo et al. 2021 [71]	Mice (C57BL/6J)	EAU	VHH	STAT3	Systemic (i.p.)	10 mg/kg twice daily, day −1 to 12	Reduced retinal inflammation on fundoscopy, OCT and histology. Preservation of ERG responses. Decreased Th17/Th1 responses and percentage of IL-17 and IFNγ expressing T cells.	No dedicated immunogenicity or toxicology assessment
Liu et al. 2022 [77]	Mice (C57BL/6)	*Aspergillus fumigatus* keratitis	VNAR	Dectin-1	Subconjunctival + topical	5 μL of 5 mg/mL subconjunctivally day 1 5 μL of 5 mg/mL topically 3 times daily from day 2	Reduced keratitis severity and clinical scores. Reduced corneal inflammatory response with decreased IL-1β and IL-6 expression.	No dedicated immunogenicity or toxicology assessment
Liu et al. 2023 [78]	Mice (C57BL/6)	*Aspergillus fumigatus* keratitis	VNAR VNAR-natamycin conjugate (Nb-NAT)	β-glucan	Subconjunctival + topical	Unspecified	Reduced fungal growth, biofilm formation, adhesion, and corneal damage. Reduced inflammatory cell infiltration and decreased IL-1β and IL-6. Nb-NAT improved antifungal efficacy.	Nb-NAT showed lower cytotoxicity and ocular irritation than natamycin
Liu et al. 2024 [79]	Mice (C57BL/6)	*Aspergillus fumigatus* keratitis	VNAR	β-glucan	Subconjunctival + topical	Unspecified	Reduced *Aspergillus fumigatus* keratitis and corneal edema. Downregulated Dectin-1, LOX-1, IL-6, IL-8, CCL2 and CXCL2. Reduced macrophage and neutrophil infiltration.	No dedicated immunogenicity or toxicology assessment
Wei et al. 2025 [80]	Mice (C57BL/6)	*Acanthamoeba* keratitis	VHH-CXCL1 fusion protein (Acab95-CXCL1)	*Acanthamoeba*-antigen	Intrastromal	1 μL (0.23 pg/μL) on day 0	Reduced *Acanthamoeba* burden and improved corneal outcome compared with free CXCL1. Slower but more sustained infiltration than CXCL alone.	High-dose free CXCL1 caused corneal damage, whereas no comparable toxicity was reported for Acab95-CXCL1
Jensen et al. 2025 [69]	Mice (C57BL/6JRj)	LI-CNV	VHH	C3 (hC3Nb1)	Subretinal	9.1 × 10^4^ IU (Lentiviral vector)	Reduction in LI-CNV size by 49% compared with irrelevant control. Reduced C3d deposition indicating inhibition of complement activation	Preserved ERG responses No anti-VHH antibodies detected in serum
Chen et al. 2026 [47]	Mice (C57BL/6J) Cynomolgus monkey	OIR LI-CNV	Bivalent VHH (humanized) Delivered using proprietary penetratin analog formulation	VEGF-A	Topical	3 μL (20 mg/mL), 3 times daily from postnatal day 12 to 16. 30 μL (1.2 mg), 3 times daily for 30 days	Significant reduction in neovascularization and avascular areas. Significant reduction in neovascularization and vascular leakage.	No treatment-related ocular or systemic toxicity Preserved ERG and no adverse effect on tissue integrity or physiological parameters
Wu et al. 2026 [55]	Mice (C57BL/6)	STZ DM	Bispecific VHH	VEGF-A, TNFα	Topical	0.2 mg/mL eye drops	Reduced retinal VEGF-A and TNFα levels. Inhibition of neovascularization and retinal structural damage.	No dedicated immunogenicity or toxicology assessment

Abbreviations: ADAs, anti-drug antibodies; C3, complement component 3; CXCL1, C-X-C motif chemokine ligand 1; EAU, experimental autoimmune uveitis; ERG, electroretinography; Fc, fragment crystallizable; ICOSL, inducible T-cell co-stimulator ligand; IL, interleukin; i.p., intraperitoneal; IU, infectious units; LI-CNV, laser-induced choroidal neovascularization; OCT, optical coherence tomography; OIR, oxygen-induced retinopathy; STAT3, signal transducer and activator of transcription 3; STZ DM, streptozotocin-induced diabetes mellitus; TNFα, tumor necrosis factor alpha; VEGF-A, vascular endothelial growth factor A; VHH, variable heavy domain of heavy chain; VNAR, variable new antigen receptor.

## Data Availability

No new data were created or analyzed in this study. Data sharing is not applicable to this article.

## Supplementary Materials

The following supporting information can be downloaded at: https://www.mdpi.com/article/10.3390/ijms27115080/s1.

## Abbreviations

The following abbreviations are used in this manuscript:

AAV	Adeno-associated viral vector
Ab	Antibody
ADA	Anti-drug antibodies
AMD	Age-related macular degeneration
ANG2	Angiopoietin-2
C	Carboxy-terminus
CDR	Complementarity-determining region
CH	Constant domain of the heavy chain
CL	Constant domain of the light chain
CNAR	Constant domain of IgNAR
CNV	Choroidal neovascularization
EAU	Experimental autoimmune uveitis
ERG	Electroretinography
Fab	Fragment antigen-binding
FDA	The U.S. Food and Drug Administration
Fc	Fragment crystallizable
FR	Framework region
hcAb	Heavy-chain-only antibody
HV	Hypervariable region
ICOSL	Induced costimulatory ligand
IgG	Immunoglobulin G
IgNAR	Immunoglobulin new antigen receptor
IL	Interleukin
IL-1β	Interleukin-1 beta
IL-6	Interleukin-6
IL-8	Interleukin-8
i.p.	Intraperitoneal
LOX-1	Lectin-like oxidized low-density lipoprotein receptor-1
LV	Lentiviral vector
mAb	Monoclonal antibody
nAMD	Neovascular age-related macular degeneration
NHP	Non-human primate
OCT	Optical coherence tomography
OIR	Oxygen-induced retinopathy
RPE	Retinal pigment epithelium
scFab	Single-chain Fab
scFv	Single-chain variable fragment
sdAb	Single-domain antibody
STAT3	Signal transducer and activator of transcription 3
TCE	T-cell epitope
TNFα	Tumor necrosis factor alpha
VEGF	Vascular endothelial growth factor
VEGFA	Vascular endothelial growth factor A
VEGFR	Vascular endothelial growth factor receptor
VH	Variable domain of the heavy chain
VHH	Variable heavy domain of heavy chain
VL	Variable domain of the light chain
VNAR	Variable new antigen receptor
