# Supplementary material for: The Promise of Single-Domain Antibodies as Ocular Therapeutics: A Narrative Review
Source: Int J Mol Sci. 2026 Jun 4;27(11):5080. doi: 10.3390/ijms27115080 (PMC13257686; doi:10.3390/ijms27115080)
Supplement: Supplementary file 1 [file ijms-27-05080-s001.zip › ijms-4310656-supplementary.pdf]

Search strategy

We searched PubMed (without publication date restriction). The final search was performed on March 13, 2026, using Google Chrome Version 145.0.7632.117 (Official Build) (64-bit)

("Single-Chain Antibodies"[Mesh] OR "Single-Domain Antibodies"[Mesh] OR Nanobod*[Text word] OR “VHH”[Text word] OR “VNAR”[Text Word] OR "Single domain antibod*" OR "Single-domain antibod*"[Text word] OR "Single chain antibod*"[Text word] OR "Single-chain antibod*"[Text word] OR “domain antibod*”)

AND

("Eye Diseases"[Mesh] OR “ocular”[Text word] OR “Eye”[Text word] OR “Retina*”[Text word] OR “Cornea”[Text word] OR “Retinopathy”[Text word] OR “Age-related macular degeneration”[Text word] OR “uveitis”[Text word])

NOT

("comment"[Publication Type] OR "editorial"[Publication Type] OR "letter"[Publication Type] OR "review"[Publication Type])

AND

("english"[Language])

Inclusion criteria:

Animal models of ocular disease

Application of single-domain antibodies as therapy

Exclusion criteria:

in vitro and ex vivo models

Comments, editorials, letters and reviews (should be excluded by the search)

Non-English (should be excluded by the search)

Abstract only

The search retrieved 104 results. The records were independently screened (full text) by two authors (T.S.J and K.K), both identifying the same 12 studies for inclusion.
